# Evolution of class 1 integrons: Mobilization and dispersal via food-borne bacteria

**DOI:** 10.1371/journal.pone.0179169

**Published:** 2017-06-06

**Authors:** Timothy M. Ghaly, Louise Chow, Amy J. Asher, Liette S. Waldron, Michael R. Gillings

**Affiliations:** Department of Biological Sciences, Macquarie University, Sydney, New South Wales, Australia; University of Manchester, UNITED KINGDOM

## Abstract

Class 1 integrons have played a major role in the global dissemination of antibiotic resistance. Reconstructing the history of class 1 integrons might help us control further spread of antibiotic resistance by understanding how human activities influence microbial evolution. Here we describe a class 1 integron that represents an intermediate stage in the evolutionary history of clinical integrons. It was embedded in a series of nested transposons, carried on an IncP plasmid resident in *Enterobacter*, isolated from the surface of baby spinach leaves. Based on the structure of this integron, we present a modified hypothesis for integron assembly, where the ancestral clinical class 1 integron was captured from a betaproteobacterial chromosome to form a Tn*402*-like transposon. This transposon then inserted into a plasmid-borne Tn*21*-like ancestor while in an environmental setting, possibly a bacterium resident in the phyllosphere. We suggest that the *qacE* gene cassette, conferring resistance to biocides, together with the mercury resistance operon carried by Tn*21*, provided a selective advantage when this bacterium made its way into the human commensal flora via food. The integron characterized here was located in Tn*6007*, which along with Tn*6008*, forms part of the larger Tn*6006* transposon, itself inserted into another transposable element to form the Tn*21*-like transposon, Tn*6005*. This element has previously been described from the human microbiota, but with a promoter mutation that upregulates integron cassette expression. This element we describe here is from an environmental bacterium, and supports the hypothesis that the ancestral class 1 integron migrated into anthropogenic settings via foodstuffs. Selection pressures brought about by early antimicrobial agents, including mercury, arsenic and disinfectants, promoted its initial fixation, the acquisition of promoter mutations, and subsequent dissemination into various species and pathogens.

## Introduction

Class 1 integrons are genetic elements that play a major role in the global dissemination of antibiotic resistance because they can capture gene cassettes from a vast pool of resistance genes [1], and are resident on diverse mobile elements [2]. All class 1 integrons possess *intI1*, a gene that encodes a site-specific recombinase (IntI1), responsible for the insertion and excision of exogenous gene cassettes at the integron-associated recombination site (*attI1*). The gene cassettes within the integron array are transcribed from a promoter (Pc) located within the coding sequence of *intI1*. This gene capture system allows generation of genomic complexity and acquisition of adaptive phenotypes [3], including resistance to nearly all known classes of antibiotics [4].

The class 1 integrons that are widely distributed in pathogens from clinical settings (hereafter referred to as ‘clinical’ class 1 integrons) are part of a more diverse group of class 1 integrons found on the chromosomes of environmental bacteria [5]. Clinical class 1 integrons are found embedded in transposons and conjugative plasmids, allowing their rapid dissemination via lateral gene transfer. As a consequence, class 1 integrons have spread to nearly all species of Gram-negative pathogens [6]. Since the clinical class 1 integron has played such a major role in the global spread of multi-drug resistance, it is important to reconstruct its evolutionary history so we can better mitigate antibiotic resistance, and gain insights into how human activities influence bacterial evolution.

It is likely that a single environmental class 1 integron gave rise to the ancestor of clinical class 1 integrons, since these all share a highly conserved *intI1* sequence [7]. In turn, this implies that a single event resulted in the movement of one variant of the class 1 integron into the human microbiota. The descendants of this initial event have given rise to a pool of genetic elements that have successfully spread into diverse bacterial species, and are now universally present in the commensal bacteria of humans and their domesticated animals [6, 8, 9]. Once this ancestral integron made its way into the human commensal or pathogenic flora, it was exposed to various selection pressures, eventually leading to the acquisition of more than 130 different resistance genes [4, 10].

The current model for the evolution of clinical integrons, however, does not answer how the pre-clinical form made its way into the human microbiota, what bacterial host facilitated this transition, or present a clear order of the complex rearrangements that lead to its clinical form, particularly, its association with the mercury resistance Tn*501*-like transposon [11]. The most likely route for movement of class 1 integrons from natural environments into the human microbiota is via water or food-borne bacteria. In particular, bacteria that occur on foodstuffs that are eaten raw, or lightly cooked, are likely candidates.

To investigate this possibility, we examined various foods for their carriage of integron-bearing bacteria, so that we can better understand the evolutionary history of the clinical class 1 integron. Here, we report: screening of baby spinach leaves from retail outlets for class 1 integrons; characterization of these integrons, their gene cassettes, and the mobile elements they reside upon; and how this information helps to inform our understanding of the events leading to the fixation of clinical class 1 integrons in the human microbiota.

## Materials and methods

### Isolation of single colonies and DNA extraction

Commercial baby spinach leaves were obtained from retail outlets in Sydney, NSW, Australia. Bacteria were isolated from leaves using a stomacher (BagMixer 400W, Interscience, St. Nom, France). Mixed cultures were screened for *intI1* by PCR, and positive cultures were plated out to obtain single colonies, which were re-screened for *intI1* [12]. Genomic DNA was extracted from *intI1*-positive single colonies using bead-beating [13]. DNA yield was assessed using agarose gel electrophoresis.

### PCR amplification and analysis

The class 1 integron integrase gene was targeted using primers HS464 and HS463a [14]. Cassette arrays from pre-clinical class 1 integrons were targeted for amplification using primers MRG284 and MRG285 [15], which amplify the region between *intI1* and immediately after the most distal *attC* site in the cassette array. Clinical class 1 cassette arrays were targeted with the primers HS458 and HS459 [14], which amplify the region between *intI1* and the 3’ conserved segment (3’-CS) [16]. To identify integron-positive isolates to species, PCRs targeted 16S rDNA with primers f27 and r1492 [17], the RNA polymerase beta subunit gene (*rpoB*) using primers RpoB-F and RpoB-R, and the 60 kDa heat shock protein gene (*hsp60*) with primers Hsp60-F and Hsp60-R [18]. PCRs targeting the plasmid partitioning gene, *parA*, were used to detect the integron-bearing plasmid, which was characterized via whole genome sequencing of *Enterobacter cloacae* isolate MN73R (described below). Primers par-1 and par-2 were designed to amplify the complete gene (S1 Table).

All PCRs were performed in 50 μL reactions containing 100 ng DNA, 25 μL GoTaq White (Promega, Madison, WI, USA), 0.5 μL of 1 mg/mL RNase and 0.5 μL of each primer (50μM). PCRs were carried out using an Eppendorf Mastercycler Epigradient S thermocycler with the appropriate thermal cycling program. PCR efficiency was assessed using 1–2% agarose gels.

Restriction digests (*Hinf* I) were performed on the MRG284/285 amplicons. All reactions were performed according to the manufacturer’s instructions (Promega) and were left to incubate overnight at 37°C. Analysis of digests was performed on 2% agarose gels, cast and run in TBE buffer [19], and post-stained with GelRed (Biotium, Fremont CA USA).

### DNA sequencing and analysis

PCR products were purified using ExoSAP-IT (Affymetrix, Santa Clara, CA, USA) as per the manufacturer’s instructions. Sanger dideoxy sequencing was carried out using the amplification primers at the Macrogen sequencing facility (Seoul, South Korea). Sequence alignments were made using Geneious v 9.0.4 software. Sequences were interrogated by searching against nucleotide databases using blastn algorithms (http://www.ncbi.nlm.nih.gov/BLAST/).

Whole-genome sequencing was carried out for one *intI1*-positive *E*. *cloacae* isolate (MN73R). Genomic DNA extracted from MN73R was sequenced on an Illumina HiSeq platform at the Garvan Institute of Medical Research (Sydney, Australia). Initial scaffolding was assembled using the A5 pipeline [20, 21], and ordered in Mauve v. 20150226 software against the *E*. *cloacae* subsp. *cloacae* ATCC 13047 reference genome (accession number NC_0144121). DNA sequences described in this study were deposited in GenBank as accession numbers KY126369-KY126372 and NFUM00000000.

## Results

### Class 1 integron screening

Washings from fresh spinach leaves were used to establish mixed cultures that were screened for *intI1* using PCR. Positive cultures were then plated to recover individual colonies. Seventy individual colonies recovered from a single collection sample were PCR positive for *intI1*. The cassette arrays of all 70 *intI1*-positive isolates were successfully amplified using primers MRG284/285 (1285 bp amplicon), but could not be amplified using HS458/459, indicating all isolates carried a pre-clinical form of the class 1 integron, prior to the formation of the 3’-CS [22]. The restriction patterns of *Hinf* I digested MRG284/285 amplicons were identical, suggesting the isolates all carried the same cassette array.

### Species identification

On the basis of 16S rDNA restriction digests and DNA sequencing of a subset of the isolates (n = 50), all integron-positive isolates were identified as *E*. *cloacae*. After further DNA sequencing of *rpoB* and *hsp60* amplicons, these isolates were subsequently identified as *E*. *cloacae* subsp. *cloacae*. To further analyse the present *E*. *cloacae* strain, the two largest assembled contigs (994 626 bp and 835 901 bp, respectively) were used in a BLAST search of *E*. *cloacae* genotypes. The top BLAST alignment for both contigs matched with an endophytic *E*. *cloacae* strain isolated from a pepper plant (accession number CP003737) [23].

### Integron characterization

**Fig 1:**
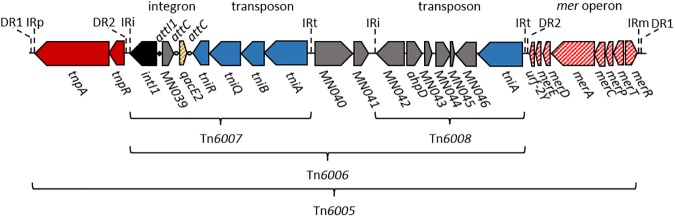
Genomic landscape of the class 1 integron reported in the present study. From left to right, the components are as follows: the direct repeat (DR1) formed by the insertion of Tn*6005*; the inverted repeat for Tn*6005* (IRp); Tn*21*-like transposition genes *tnpA* and *tnpR*; the direct repeat (DR2) formed by the insertion of Tn*6006*; the inverted repeat for Tn*6006*/*6007* (IRi); a class 1 integron with *intI1* and integron-associated recombination site *attI1*, carrying two gene cassettes, *MN039* and *qacE2*, each with a cassette-associated recombination site *attC*; Tn*402*-like transposition genes *tniR*, *tniQ*, *tniB*, *tniA*; the inverted repeat for Tn*6007* (IRt); genes *MN040* and *MN041*; the inverted repeat for Tn*6008* (IRi); the *Tn6008* transposon, carrying genes *MN042*, *ahpD*, *MN043*, *MN044*, *MN045*, *MN046*, and *tniA*; the inverted repeat for Tn*6006*/*6008* (IRt); the direct repeat (DR2) formed by the insertion of Tn*6006*; Tn*21*-like *mer* operon consisting of *urf-2Y*, *merE*, *merD*, *merA*, *merC*, *merP*, *merT* and *merR*; the inverted repeat for Tn*6005* (IRm); and the direct repeat (DR1) formed by the insertion of Tn*6005*. This whole element is embedded in an IncP plasmid whose sequence is lodged as accession number KY126370.

DNA sequencing of *intI1* and cassette arrays revealed an integron carrying two gene cassettes, neither of which carried a typical cassette-encoded antibiotic resistance gene (Fig 1). The integron did not contain the 3’ conserved segment (*qacEΔ1* / *sul1*) usually found in clinical class 1 integrons, suggesting this was a pre-clinical integron. All isolates had an identical integron-integrase sequence, which displayed 99.96% sequence identity (1 bp difference) with a class 1 integron (accession number EU591509) found in *E*. *cloacae* isolated from human feces [24]. The first cassette contained a gene designated as *MN039*, whose predicted gene product is an NADPH-dependent flavin mononucleotide (FMN) reductase, an enzyme family responsible for the reduction of quinones. The second cassette contained *qacE2*, whose product is an efflux pump that can confer resistance to biocides such as antiseptics and disinfectants [25].

**Fig 2:**
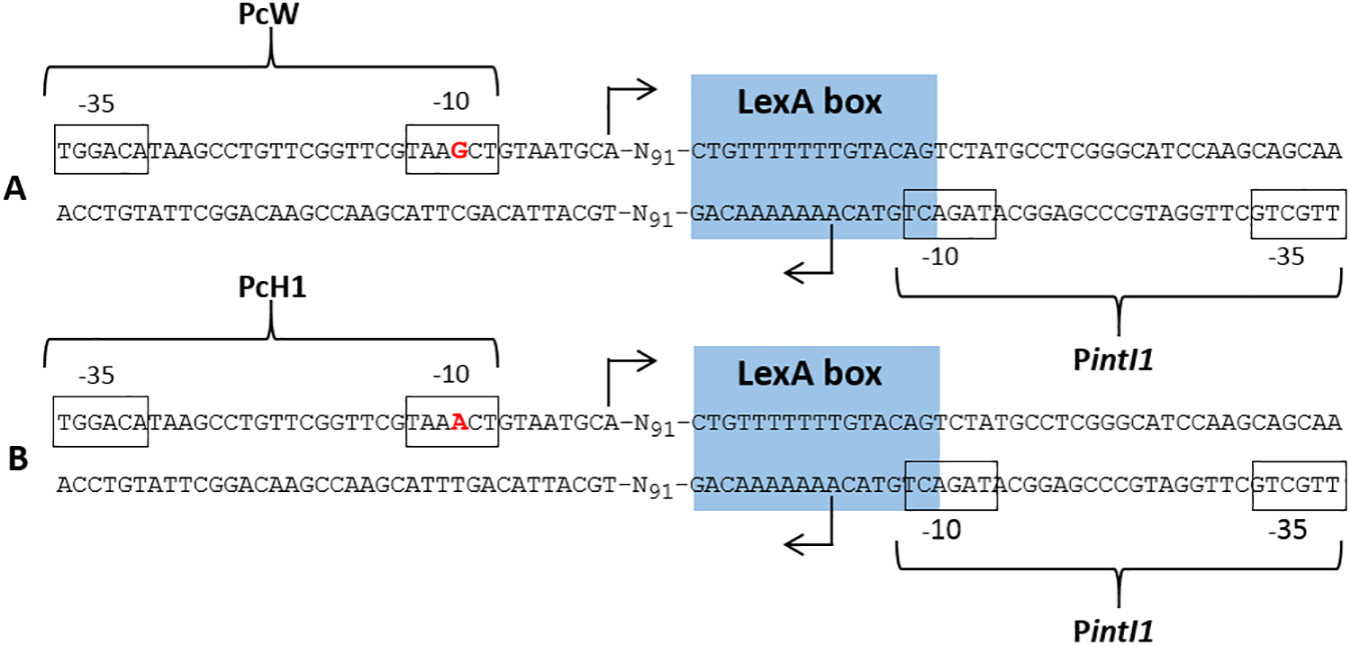
Detailed structure of the class 1 integron promoter regions. The -35 and -10 motifs for the Pc and P*intI1* promoters are boxed; a point mutation in the -10 motif, which distinguishes the PcW and PcH1 promoters, is highlighted in red; the transcription initiation sites are indicated by arrows; the LexA box is shaded in blue (expression of *intI1* is regulated by the SOS response). (A) Promoter region within the class 1 integron described in the present study, containing the PcW promoter variant, which is also present in a number of chromosomal class 1 integrons (accession numbers EU316185 and EU327987-EU327991). This strongly suggests PcW is the ancestral promoter in these integrons; (B) Promoter region within the otherwise identical class 1 integron characterized from the human fecal flora by Labbate et al. (23), which contains the PcH1 promoter.

Sequence comparison between the present integron and that previously characterised by Labbate et al [24], showed a single base pair difference in the promoter region, Pc (Fig 2). Four predominant Pc variants occur in class 1 integrons, based on the sequence of their -35 and -10 hexamer motifs. The integron described here had the PcW (weak) promoter, thought to be the ancestral promoter type [26, 27]. The integron described by Labbate et al [24] contained the PcH1 (hybrid 1) promoter (Fig 2).

### Genomic landscape of the integron

To understand the evolutionary history of the integron and its potential for lateral transfer, we used the genome sequence to reconstruct the genetic landscape around the integron. The integron was located within a Tn*402*-like transposon, designated Tn*6007* (Fig 1) [24]. The transposons Tn*6007* and Tn*6008* possess homologous pairs of inverted repeats (IRi/IRt), and together form Tn*6006* [24]. The complete Tn*6006* module appears to have transposed as a single unit. This can be inferred by the flanking direct repeats (DR2), which indicate a duplication at the insertion site. Here, Tn*6006* was inserted into the *res* site of a Tn*501*-like transposon, which also carried the *mer* operon. The Tn*501*-like transposon, together with the Tn*6006* insert, formed Tn*6005* (Fig 1) [24]. The Tn*6005* compound transposon was itself located within a 114 969 bp plasmid, designated pOP-I. The plasmid, whose complete sequence is lodged under accession number KY126370, carried a type IV conjugation system. The plasmid also carried the DNA-binding protein gene *kfrA*, which binds to the replication region of IncP-1 plasmids [28]. We therefore designate pOP-I as part of the IncP family of broad-host range plasmids. PCR screening and Sanger sequence analysis of *parA* confirmed the presence of pOP-I in all the *intI1*-positive isolates tested in this study.

## Discussion

Class 1 integrons have played a major role in spreading multidrug-resistance in bacteria [29]. However, the clinical class 1 integrons responsible for this dissemination are just one sequence type in a larger family of class 1 integrons that are broadly distributed in environmental bacteria, and which exhibit considerable sequence diversity [5, 11]. Almost without exception, the DNA sequence of the integrase gene (*intI1*) of all clinical class 1 integrons is identical [7]. Given the extensive sequence diversity in this family of elements, the sequence identity amongst clinical class 1 integrons is strong evidence that they have a single, recent common ancestor. Understanding the origins of clinical class 1 integrons and the reasons for their spectacular success will help us understand the dynamics of antibiotic resistance, and the influence that humans have had on the bacterial resistome [30].

Here, we identified a class 1 integron that is a strong candidate for the type of element that might be the common ancestor of the clinical class 1 integrons. The integron was located on the conjugative IncP family plasmid, pOP-I, resident in *E*. *cloacae* subsp. *cloacae* isolated from the phyllosphere of baby spinach leaves. Various characteristics of this class 1 integron and its genetic landscape agree with the properties expected of the immediate precursor to the clinical class 1 integron, as outlined below.

The integron described here was inserted into a Tn*402*-like transposon, and was linked to another transposon, Tn*6008*, to form the composite transposon Tn*6006* (Fig 1) [24]. The complete Tn*6006* module has then inserted at the *res* site of a Tn*501*-like transposon (Tn*21* ancestor), carrying a *mer* operon (Fig 1) to generate transposon Tn*6005*, previously described by Labbate et al. [24]. Here, this entire construct was recovered from an environmental bacterium, and consequently appears to predate the infiltration of this kind of element into clinical environments.

All integrons of clinical importance (classes 1, 2 and 3) have inserted into transposable elements, thus markedly increasing their potential for mobility [10, 31]. The immediate ancestor of the clinical class 1 integron is a good example, and is thought to have arisen when a chromosomal integron from an environmental betaproteobacterium inserted into a transposon. This conclusion is based on the discovery of chromosomal integron-integrase genes (*intI1*) with sequence identity to those now seen in clinical integrons [11]. This ancestor was initially mobilized into a *res* hunting transposon of the Tn*402* type [32], and consequently we would expect this first molecular event to result in a clinical *intI1* sequence attached to a complete set of Tn*402* transposition genes, as seen here. Further, chromosomal integrons carry gene cassettes unrelated to the antibiotic resistance cassettes found in contemporary clinical integrons, and this should also be the case in the class 1 ancestor. The integron we describe here was linked to complete Tn*402* transposition machinery, lacked the 3’-CS indicative of clinical forms [22], and carried two non-antibiotic resistance cassettes (Fig 1), as predicted for the model clinical ancestor.

The first of these cassettes (*MN039*), encoded a predicted NADPH-dependent FMN reductase. Enzymes in this family can provide resistance to toxic substances, such as arsenic [33] and other oxidative stressors [34]. The second cassette carried a gene encoding an efflux pump, QacE2. The *qacE2* gene product is an efflux pump for cationic compounds, thus also providing some resistance to disinfectants and antiseptics [25]. Cassettes encoding Qac efflux pumps are common in environmental class 1 integrons, and have been predicted to be part of the earliest forms of clinical class 1 integrons [15], in keeping with expectations for a clinical ancestor.

These observations present an insight into the evolutionary history of clinical class 1 integrons. Transposon Tn*21* and its various derivatives have had a central involvement in the global dissemination of antibiotic resistance determinants. It was previously thought that the ancestral class 1 integron inserted into the ancestral *mer* transposon after partial deletion events of the *qacE* cassette and *tni* module, using transposition machinery supplied in *trans* [35]. Here we suggest an alternate order of assembly, whereby the pre-clinical integron within the *cis*-acting Tn*402* inserted into the Tn*21* ancestor prior to any deletion events. This would also suggest that the formation of Tn*21* occurred in an environmental setting. Under this interpretation, the integron we describe here represents an intermediate stage in the complex rearrangements that preceded the dissemination of clinical class 1 integrons into the human microbiota.

The occurrence of the present integron in an *E*. *cloacae* strain isolated from baby spinach leaves, provides a plausible route for transmission of environmental integrons into the human microbiota. *E*. *cloacae* is often found associated with plants, and can be readily isolated from spinach leaves [36], also being found as an endophyte in spinach xylem vessels [37]. The consumption of uncooked spinach leaves therefore provides a plausible mechanism for transfer of *E*. *cloacae* carrying the pre-clinical class 1 integron into the human gut. Bagged, ready to eat spinach has been shown to be a vehicle for transmission of Gram negative bacteria into the human microbiota [38], and *E*. *cloacae* itself is an inhabitant of the digestive tract, where it is a commensal organism [39].

This integron is known to have made the transition into the human gut because the same element has previously been described from human fecal flora [24]. Although it is possible that this is a result of human contamination of agricultural plants, we suggest two reasons why this is unlikely. First, *rpoB* sequence analysis shows the present *E*. *cloacae* isolate to be representative of environmental strains, isolated from endophytic and soil environments (e.g. accession numbers CP003737, JN627207, LC049166). Furthermore, the two largest assembled contigs (994 626 bp and 835 901 bp, respectively) of the sequenced strain, both exhibited a top BLAST alignment (99% identity) with an enodphytic *E*.*cloacae* strain isolated from a pepper plant (accession number CP003737) [23]. These sequence alignments suggest that the present isolate is an environmental strain associated with plants, and is unlikely to be a human contaminant.

The second, a single nucleotide variation in the cassette promoter region (Pc), distinguishes the two integrons (Fig 2). There are four main Pc variants defined on the basis of their -35 and -10 hexamer motifs, each with different transcriptional strengths: PcW; PcH1; PcH2; and PcS [26]. There is a 30-fold increase in strength from the weakest promoter, PcW, to the strongest promoter, PcS [40]. The integron in the present study had the PcW variant, which is thought to be the ancestral form, while that characterized by Labbate et al [24] contained PcH1. This change in promoter sequence does not affect the IntI1 amino acid sequence (due to a silent base substitution; Fig 2), and thus does not change IntI1 excision activity [41]. However, PcH1 is associated with a 4.5-fold higher level of cassette expression [40]. Thus, conversion of PcW to PcH1 enhances gene cassette transcription without restricting the capacity for cassette reorganization. Such a mutation is believed to have occurred early in the history of clinical integrons [40]. In support of this, the PcH1 variant is commonly found among clinical forms of class 1 integrons (e.g. accession numbers KX169264, KP099552, LC169585, LC169566, and CP014662), suggesting that this point mutation occurred in anthropogenic settings. Therefore, we propose that the present integron, containing the PcW variant, was the precursor to the integron containing the PcH1 variant, and that this promoter variant was subsequently selected to provide enhanced cassette expression in the environment of the human gut. Hence, the present integron, as well as its bacterial host, appear to have environmental origins, and their occurrence on spinach leaves is unlikely to be a consequence of human contamination.

**Fig 3:**
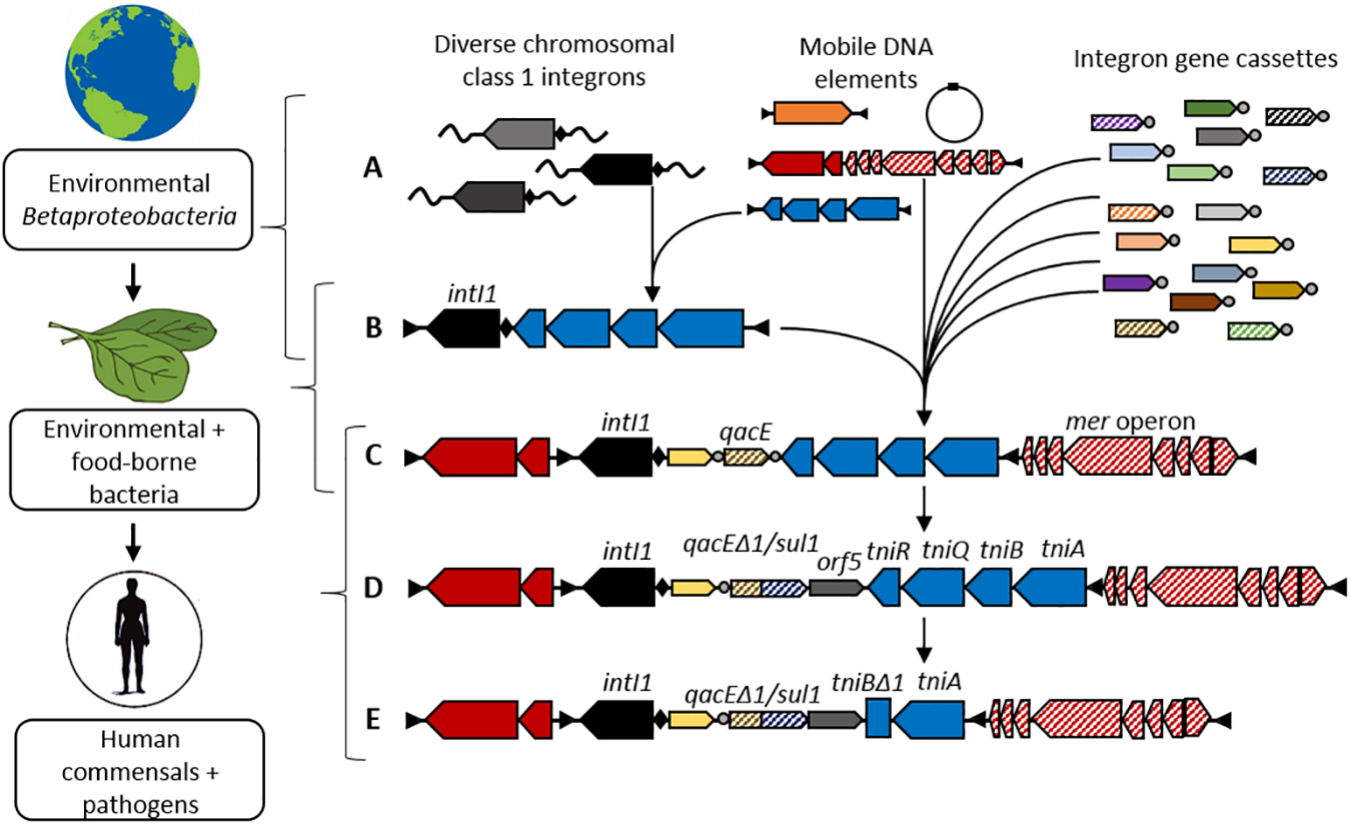
A model for the origin of clinical class 1 integrons. (A) Diverse chromosomal class 1 integrons that are present in environmental *Betaproteobacteria* can interact with mobile DNA elements such as transposons and plasmids in the environment. Integrons have access to a vast pool of integron gene cassettes including the *qacE* cassette that encodes a membrane efflux pump; (B) A single chromosomal integron is captured by a Tn*5090*-like transposon, to generate Tn*402*; (C) Now mobilized, the integron is free to move between a range of bacterial species. In particular, the Tn*402* transposon inserts into the mercury resistance Tn*501*-like transposon, to generate Tn*21*. Residence of this complex DNA element on a broad host range plasmid allows the integron to make its way into the human commensal flora via food-borne bacteria; (D) Once resident within the human microbiota, the integron is fixed by selection, driven by mercury and disinfectants, and after introduction of sulfonamide antibiotics, captures the *sul1* and *orf5* gene cassettes to delete part of the original *qacE* cassette; (E) Partial deletions of the *tni* module, and the collective acquisition of diverse resistance cassettes, lead to the diversity of clinical class 1 integrons that have since disseminated around the globe.

While we will never know the precise series of events that generated the clinical class 1 integron, we can now reconstruct a plausible scenario based on the properties of the integron we describe here. One variant of the diverse betaproteobacterial chromosomal class 1 integrons (Fig 3A), carrying a *qac* gene cassette, was captured by a Tn*5090*-like transposon, to form Tn*402* (Fig 3B). This hybrid element targeted the *res* site of a plasmid bearing the mercury-resistance Tn*501*-like transposon, to generate the compound transposon Tn*21* (Fig 3C) [35]. The location of this complex mosaic element on a broad host range IncP plasmid would then have allowed its conjugative transfer into a diversity of bacterial hosts.

The subsequent history of the clinical class 1 integron involves molecular diversification and dissemination into diverse hosts. Introduction of the first true antibiotics in the 1930s, the sulfonamides [42], would have selected for acquisition of the resistance gene *sul1*, at the same time deleting part of the *qacE* gene cassette to generate the 3’ conserved segment [22], which is now a feature of many clinical class 1 integrons (Fig 3D). Various deletions also occurred in the *tni* transposition module (Fig 3E). These events, plus the collective acquisition of over 130 antibiotic resistance gene cassettes [4] have generated the diverse structures of contemporary clinical class 1 integrons (Fig 3E).

In summary, we have demonstrated the environmental occurrence of a class 1 integron that resides within a complex set of transposons and is capable of being mobilized by a broad host range IncP plasmid. This DNA element was found in a bacterial species that can colonize both plants and the human gut, and it was recovered from a plant food that can be consumed raw, thus providing a highly plausible route into the human microbiota. Once resident in the microbiota, the possession of genes known to confer resistance to arsenic, mercury and disinfectants supply both the integron and its bacterial host with a means of preferential survival, since all these agents of selection were in use well before the antibiotic era.

From a single molecular rearrangement that happened in a single cell perhaps as recently as 100 years ago, this one sequence variant of the class 1 integron has vastly increased in abundance and distribution. Its spectacular success has been driven by population expansion of humans and their domestic animals, overlaid by the intense selection driven by antimicrobial agents.

## Supporting information

S1 Table PCR primers and annealing temperatures used in this study. (DOCX)
