# Supplementary material for: Evolution of class 1 integrons: Mobilization and dispersal via food-borne bacteria
Source: PLoS One. 2017 Jun 6;12(6):e0179169. doi: 10.1371/journal.pone.0179169 (PMC5460862; doi:10.1371/journal.pone.0179169)
Supplement: S1 Table — (DOCX) [file pone.0179169.s001.docx]

| *Target* | *Primers* | *Sequence (5’-3’)* | *Annealing temperature* | *Product size (bp)* | *Reference* |
| --- | --- | --- | --- | --- | --- |
| *intI1* | HS464  HS463a | ACATGCGTGTAAATCATCGTCG  CTGGATTTCGATCACGGCACG | 60°C | 473 | (14) |
| Pre-clinical class 1 integron cassette array | MRG284 MRG285 | GTTACGCCGTGGGTCGATG  CCAGAGCAGCCGTAGAGC | 60°C | Variable* | (15) |
| Clinical class 1 integron cassette array | HS458  H459 | GTTTGATGTTATGGAGCAGCAACG  GCAAAAAGGCAGCAATTATGAGCC | 60°C | Variable* | (14) |
| 16S rDNA | f27  r1492 | AGAGTTTGATCMTGGCTC  TACGGYTACCTTGTTACGACTT | 60°C | 1379 | (17) |
| *rpoB* | RpoB-F  RpoB-R | AACCAGTTCCGCGTTGGCCTGG  CCTGAACAACACGCTCGGA | 58°C | 973 | (18) |
| *hsp60* | Hsp60-F  Hsp60-R | GGTAGAAGAAGGCGTGGTTGC  ATGCATTCGGTGGTGATCATCAG | 58°C | 330 | (18) |
| *parA* | par-1  par-2 | CTTCAGCTTGTTCTGCCG  CAAGCATTGACTATAACTGCG | 50°C | 772 | Present study |

**S1 Table**. PCR primers and annealing temperatures used in this study.

* PCR amplicon size will vary depending on the number and size of integron-associated gene cassettes.
